# CHIPOFIL: A pilot study assessing the feasibility of HIPEC without extracorporeal circuit

**DOI:** 10.1515/pp-2019-0008

**Published:** 2019-07-26

**Authors:** Pablo Ortega-Deballon, Olivier Facy, Christine Binquet, Delphine Delroeux, Patrick Rat

**Affiliations:** Digestive Surgical Oncology, Equipe Avenir, 14 rue Paul Gaffarel, Dijon 21079, France; Digestive Surgical Oncology, University Hospital of Dijon, Dijon, France; CIC Epidémiologie Clinique, INSERM CR 1231, Dijon, France; Digestive Surgical Oncology, University Hospital of Besançon, Besançon, France; Service de Chirurgie Digestive et Cancérologique, CHU Bocage Central, 14, rue Paul Gaffarel, Dijon Cedex 21079, France

**Keywords:** heated intraperitoneal chemotherapy, heating wire, HIPEC, hyperthermia, peritoneal carcinomatosis

## Abstract

**Background:**

Heated intraperitoneal chemotherapy (HIPEC) is currently performed using an external circuit including a heating device and a pump. Available devices have several drawbacks in terms of costs, technique (flow surges due to blocked tubes) and staff safety, hindering a wider use. In a previous preclinical study conducted in animals, we placed a heating wire within the abdomen to achieve and maintain hyperthermia. Our results showed this technique is safe and effective. The present pilot study was conceived as the first use of such a device in humans, aiming to confirm its safety and efficacy.

**Methods:**

This was a pilot study designed to include 13 patients undergoing HIPEC. Two sets of the prototype were placed within the abdominal cavity, one in the supramesocolic and one in the inframesocolic space. The target temperature was 42–43 °C during 30–90 min according to the protocol defined for each patient. The time to set up, heat and dismantle was measured. All complications were recorded during the first postoperative year and evaluated by an independent committee.

**Results:**

Nine women and four men were included. The median time to set on the device was 25 min. The target temperature was obtained in a median of 14 min and maintained uniform and homogeneously distributed within the abdomen for the scheduled duration. A permanent stirring of the viscera was performed. No thermal injury or device-related complications were observed. There were two anastomotic leaks (only one requiring reoperation), two hemoperitoneum requiring reoperation, one evisceration and one gastroparesia.

**Conclusions:**

A heating cable within the peritoneal cavity can achieve safe, simple, fast and efficient HIPEC.

## Introduction

Complete cytoreductive surgery (CRS) followed by heated intraperitoneal chemotherapy (HIPEC) has become in recent years the standard treatment for several peritoneal malignancies. The current techniques of HIPEC over the world use a heating device, a pump and an external circuit with inflow and outflow tubes in order to heat and infuse the chemotherapy in the peritoneal cavity and maintain hyperthermia [1]. Different machines are commercially available for this purpose. All these devices have a significant cost, and this may limit the use of HIPEC in several countries and, thus, the access of patients to this therapy. Beyond the economic aspect, inflow or outflow tubes may get blocked (with temperature dropping) or leak chemotherapy liquid and require the watchful surveillance of one member of the team (in addition to the surgeon). Those drawbacks of HIPEC have probably hindered its wider use around the world.

We hypothesized that the heating source could be placed straight ahead within the abdomen. This could achieve *in situ* hyperthermia and avoid the need for the current commercially available machines with their specific drawbacks. A prototype (Thermowire^®^) consisting of a heating cable placed within the abdomen was then conceived. The safety and the efficacy of this device have been previously established in a large swine model [2, 3].

The CHIPOFIL pilot study was conceived as a prospective first use of Thermowire^®^ in humans. The first aim of the study was to establish the safety of this device for human clinical use. Secondary aims were to assess the quality of hyperthermia and the time to set up the device and achieve the target temperature.

## Patients and methods

### Design of the study

The CHIPOFIL prospective study was a pilot study assessing for the first time the safety of Thermowire^®^ in humans. All patients undergoing HIPEC were eligible, except in case of known allergy to latex due to the use of an abdominal cavity expander [4]. To calculate the sample size, a Bayesian approach was performed. Based in a risk of thermal injury similar or higher than with other techniques of HIPEC (1% per patient according to the most “pessimistic” data) and a chance of obtaining hyperthermia close to other techniques of HIPEC (90% per procedure according to the most “pessimistic data”) [5, 6, 7], the number of patients needed to obtain either a thermal injury or the impossibility to achieve hyperthermia was established at 12. In order to have 12 patients with all data available, one more patient was added in case of missing data. Thus, 13 patients were to be included in the study. Patients were included between January 2015 and June 2016 after they provided a written informed consent.

### Description of the device

**Figure 1:**
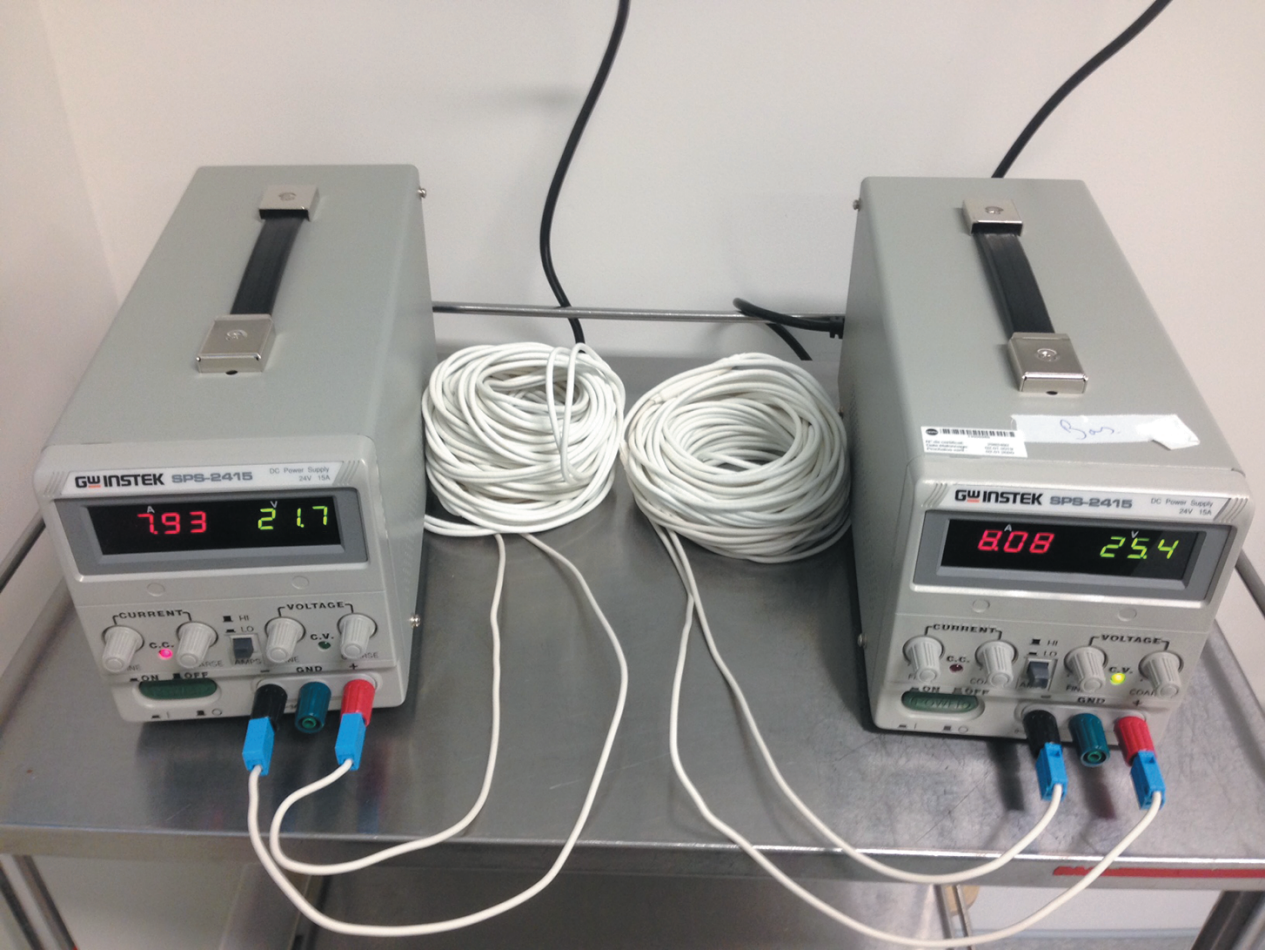
View of the 2 units of 24-V transformer and regulator and the heating cable. Several lengths of cable are available although in the present study we always used 2 sets of 17 meters of length.

The device consists of a disposable electric heating cable covered with silicone rubber insulation. This cable is connected to a 24-V transformer and regulator, which is connected to current electric outlet (Figure 1). Several lengths of cable are available for use in order to adapt to each patient’s anatomy (17 m, 13 m, 10 m, 7 m, 5 m and 3 m; references JB001 to 006, respectively). In the present study, two sets of 17 m Thermowire^®^ were used for each patient. The device undergoes sterilization with ethylene oxide by the current manufacturer (DistriClass Medical SA, Chaponnay, France). The heating cable (Thermowire^®^) is not still CE certified, while the electric transformer is already certified by the manufacturer. According to the Directive 93/42/CEE, Thermowire^®^ is a “temporary, surgically invasive, therapeutically active, single use device type IIa”.

### Surgical technique and follow-up

**Figure 2:**
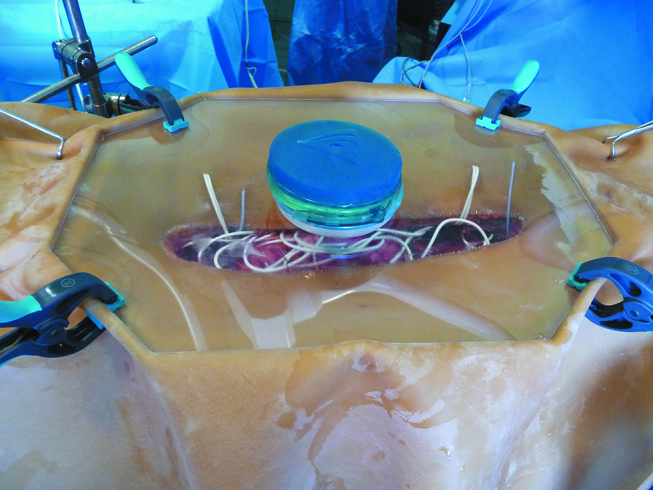
Overview of the system including the abdominal cavity expander with its cover (the « glove-box » system) and the heating wire. No pump, no circuit, no tubes are necessary anymore.

We used a large midline laparotomy to access the abdomen. After CRS was achieved in all patients, an open-abdomen closed-HIPEC technique was performed as previously described in detail elsewhere and currently performed in our department since 15 years [4]. Briefly, it consists of a latex abdominal cavity expander stapled to the cutaneous edges and suspended on a metallic frame held by a Thompson retractor (Landanger, France). A transparent methacrylate cover with a Gelport in its centre (Landanger, France) is placed over the metallic frame and the latex piece hermetically closing the abdominal cavity. Three temperature probes (one fixed with a stitch to the diaphragm, one close to the mesentery root and one into the pelvis) were passed through the latex sheet and connected to an integrated system of temperature control. The abdominal cavity was filled in with 2 L/m^2^ of physiologic serum at 37 °C. Two sets of 17 m long Thermowire (JB001) were used for every patient, one in the supramesocolic and one in the inframesocolic area (Figure 2, Video in the Supplementary Material). The loops of the heating cable passed through the abdominal cavity expander and were uniformly distributed within the abdominal cavity, between bowel loops, in the infradiaphragmatic and infrahepatic areas, in the lesser sac, in both paracolic gutters and in the pelvis. We switched on both devices and the temperature progressively increased until 42 °C was reached. The target temperature was 42–43 °C, during 30, 60 or 90 min depending on the drug used (30 min for oxaliplatin, 60 min for mitomycin C and 90 min for all other protocols). In our department, viscera are constantly stirred, whatever the HIPEC technique. At the end of HIPEC, the device was dismantled and the whole abdominal cavity contents were carefully examined searching for any thermal injury.

The postoperative surveillance was made according to the current protocols of each institution. For the purposes of the present study, the follow-up ended at 1 year of the procedure.

### Ethics and safety issues

All adverse events within 1 year after HIPEC were recorded and evaluated by an independent committee of experts composed of one surgeon and two oncologists from non-participating institutions in order to determine any potential relationship with the use of Thermowire^®^.

This protocol received approval from the French National Agency watching over the security of drugs and medical devices (ANSM), the Institutional Board Review and the Regional Ethics Committee (CPP Est 1: ID RCB N° 2012-A01509-34). The study was registered in clinicaltrials.gov (NCT02862899).

## Results

### Description of the patients

Thirteen consecutive patients (nine women and four men; median age: 59 ± 9 years) underwent HIPEC using Thermowire^®^. Their weight was 66 ± 13 kg and their body surface was 1.74 ± 0.2 m^2^. The disease leading to HIPEC was colorectal cancer in eight patients, appendiceal cancer in two and gastric cancer, pseudomyxoma peritonei and peritoneal mesothelioma in each one of the three other patients. The peritoneal carcinomatosis index (PCI) ranged between 0 and 20 (median: 6). In five patients, HIPEC was prophylactic (PCI=0). In all other patients, a CRS (CC-0) was achieved. The drugs used were mitomycin-C in seven patients, oxaliplatin in four, cisplatin and doxorubicin in one and cisplatin and mitomycin-C in one. A homogenous and constant hyperthermia was obtained in all patients, with temperatures ranging between 42 °C and 43 °C in all three thermal probes during the scheduled time according to each protocol (30, 60 or 90 min).

### Time to set up and dismantle the device

**Figure 3:**
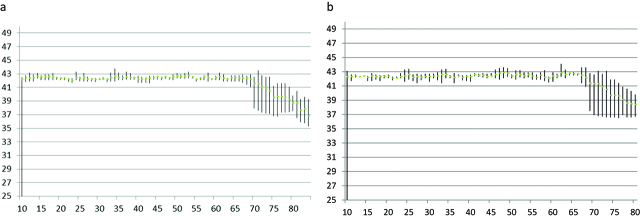
Mean temperatures (with min and max temperature) recorded at the diaphragm (a) and Douglas (b) thermal probes along 1-hour procedures of HIPEC. The curves show the thermal homogeneity achieved with the device.

The median time to set up the device (abdominal cavity expander and Thermowire^®^) was 23.7 ± 5.7 min. The target temperature was obtained in 10.3 ± 4.3 min and maintained along all the procedure homogeneously in the thermal probes. No peak or drop of temperature was registered during the desired hyperthermia period in any patient (Figure 3). The time to dismantle the device was 10.5 ± 4.2 min.

### Morbidity

Two patients presented a colorectal anastomotic leak (one requiring reoperation), two had hemoperitoneum requiring reoperation (both in oxaliplatin patients), one evisceration and one prolonged gastroparesia (resolved spontaneously at 2 months). No other complications were registered. Namely, there was no thermal injury. No complication was related to the use of Thermowire^®^ according to the scientific committee and the independent committee of the study.

## Discussion

Our results show for the first time that HIPEC can be safely performed without the use of an external circuit with inflow and outflow lines and a pump. With this new device, homogeneous and uniform intraperitoneal hyperthermia was achieved in all patients. The installation and dismantlement of Thermowire^®^ was quick and easy. Placing the heating source within the abdomen overcame all problems of tubes becoming blocked and flow surges.

The potential benefits of the use of Thermowire^®^ as compared to available pumps and external circuits are multiple. The precise reduction in costs cannot be evaluated as the device is not still commercially available, but it will be clearly less expensive than any currently available pump; this should make HIPEC more easily available everywhere, namely in developing countries. The time necessary to set up and dismantle the device reduces the duration of the HIPEC procedure, something appreciated by surgeons performing HIPEC after a long and somewhere challenging CRS time. The set-up of this device is quite simple. There are no more tubes that may obstruct stopping the circuit and leading to a drop in temperature. The absence of inflow and outflow tubes avoids additional holes across the abdominal wall. Last, but not least, avoiding an external circuit for heated chemotherapy reduces the risk of spillage of drugs and increases safety at work for all operating room staff.

The main concern with this new concept is the risk of thermal injury. Stirring within the abdominal cavity was always performed due to the fear of thermal injuries in case of a close and permanent contact between a segment of the heating cable and the viscera. But this is also our current technique in HIPEC in order to optimize heat distribution [4]. We have not evaluated Thermowire^®^ in closed HIPEC. The constant stirring of viscera allowed by open procedures might be an essential protection against thermal injuries. During our preclinical study in the pig, no life-threatening injury was observed despite a 1-h contact between the heating wire and a bowel loop around which the wire had been knotted; the three areas with an aspect of burn yielded only a prenecrotic ulceration of the mucosa and the integrity of the remaining layers of the bowel [2]. However, this is insufficient evidence to use Thermowire^®^ in closed HIPEC. A specific pilot study in this setting is warranted, probably limiting HIPEC to 30-min duration at the beginning, and going to longer durations in the absence of any injury.

Using two sets of Thermowire^®^ for the supramesocolic and the inframesocolic areas, respectively, seems important because they often behave differently with regard to temperature [3]. An independent energy management makes easier to achieve thermal homogeneity in the whole abdominal cavity.

It could be argued that the absence of an external circuit might not be an improvement because the outflow puts away from the patient the liquid containing tumour cells. To the best of our knowledge, it has never been proved that filters in the circuit retain tumour cells and could contribute to decrease the concentration of malignant cells within the peritoneum. To our knowledge, no authors have performed HIPEC without an external circuit and a pump. Cho et al. evaluated two annular-phased-array applicators in order to perform external regional hyperthermia associated with systemic standard chemotherapy for peritoneal carcinomatosis, but there was no HIPEC [8]. Mochiki et al. performed gastrectomy with postoperative intraperitoneal hyperthermochemotherapy using Thermotron RF-8 (a heating device that can raise temperatures in both superficial and deep-seated tumours using radiofrequency electromagnetic waves as a source of heat) [9]. However, the concept of heating the liquid inside the abdomen during surgery in HIPEC is totally new. This technique has become the standard for HIPEC in our department and more than 100 patients have undergone operation without any device-related injury.

We conclude that a heating cable placed within the peritoneal cavity is a safe, simple, fast and effective way to perform HIPEC. This avoids the use of any machine or external circuit with their inherent drawbacks and makes HIPEC simple and more available to new teams worldwide.
